# Supplementary material for: Human β-Defensin 2 (HBD-2) Displays Oncolytic Activity but Does Not Affect Tumour Cell Migration
Source: Biomolecules. 2022 Feb 6;12(2):264. doi: 10.3390/biom12020264 (PMC8961614; doi:10.3390/biom12020264)
Supplement: Supplementary file 1 [file biomolecules-12-00264-s001.zip › biomolecules-1504163-supplementary.pdf]

Supplementary data

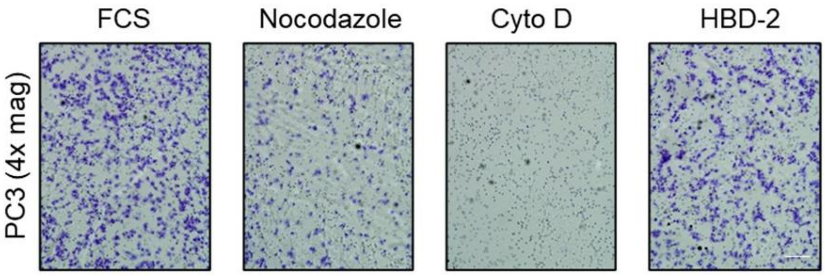

Figure S1: Microscopic images of PC3 cells taken over 6h, with FCS, nocodazole (20 μM), cytochalasin D (10 μM) and HBD-2 (5 μM). Purple stain represents migrated cells. Scale bar represent 10 μm.
